# Proteogenomics in Nephrology: A New Frontier in Nephrological Research

**DOI:** 10.3390/cimb46050279

**Published:** 2024-05-11

**Authors:** Kavya Chavali, Holley Coker, Emily Youngblood, Oleg Karaduta

**Affiliations:** 1 Adventist Health Hanford Family Medicine Residency Program, ONE Adventist Health Way, Roseville, CA 95661, USA; 2 Department of Physician Assistant Studies, University of Arkansas for Medical Sciences, 4301 W Markham Street, Little Rock, AR 72205, USA

**Keywords:** proteogenomics, proteomics, nephrology, kidney cancer, biomarkers

## Abstract

Proteogenomics represents a transformative intersection in nephrology, uniting genomics, transcriptomics, and proteomics to unravel the molecular intricacies of kidney diseases. This review encapsulates the methodological essence of proteogenomics and its profound implications in chronic kidney disease (CKD) research. We explore the proteogenomic pipeline, highlighting the integrated analysis of genomic, transcriptomic, and proteomic data and its pivotal role in enhancing our understanding of kidney pathologies. Through case studies, we showcase the application of proteogenomics in clear cell renal cell carcinoma (ccRCC) and Autosomal Recessive Polycystic Kidney Disease (ARPKD), emphasizing its potential in personalized treatment strategies and biomarker discovery. The review also addresses the challenges in proteogenomic analysis, including data integration complexities and bioinformatics limitations, and proposes solutions for advancing the field. Ultimately, this review underscores the prospective future of proteogenomics in nephrology, particularly in advancing personalized medicine and providing novel therapeutic insights.

## 1. Introduction to Proteogenomics in Nephrology

In the burgeoning field of nephrology, the advent of proteogenomics marks a paradigm shift, promising to elucidate the intricate molecular underpinnings of kidney diseases. Proteogenomics, an interdisciplinary approach, synergistically integrates the disciplines of genomics, transcriptomics, and proteomics. This convergence is instrumental in fostering a comprehensive understanding of how genetic and transcriptomic alterations are intricately linked to proteomic variations. By mapping these interrelations, proteogenomics provides an unprecedented view of the molecular landscape of biological systems and diseases [1,2].

The application of proteogenomics in nephrology is particularly pivotal. Nephrology, a field traditionally reliant on the understanding of complex renal pathophysiology, has witnessed significant advancements through proteomic studies. These studies have delved into the renal proteome, encompassing the proteomes of specific intrarenal structures such as the glomeruli, tubules, and podocytes. Furthermore, they extend to the analysis of the urinary proteome and the protein profiles in dialysate or ultrafiltrate from renal replacement therapies [1,3,4,5]. The insights garnered from these proteomic analyses have been instrumental in delineating the pathophysiological landscape of renal diseases, identifying novel biomarkers, and uncovering new therapeutic targets.

Incorporating proteogenomics into this framework enhances the granularity and accuracy of these insights. It facilitates the identification of novel gene isoforms, splice variants, and post-translational modifications which are critically involved in renal pathologies [6,7]. Moreover, it aids in elucidating the molecular mechanisms underpinning renal diseases. This comprehensive molecular characterization is particularly crucial in the context of complex kidney diseases like CKD, where proteogenomics can uncover molecular alterations associated with pathophysiological processes such as fibrosis.

**Figure 1:**
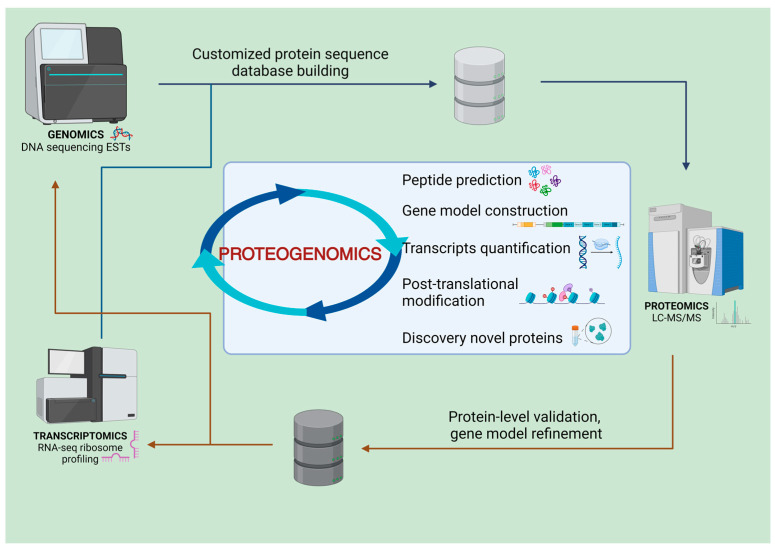
In proteogenomics, a blend of genomic and transcriptomic information is utilized to create custom protein databases, enhancing the interpretation of proteomic data. This method allows for the validation of gene expression at the protein level and aids in refining gene models. Improved gene models, in turn, contribute to the advancement of protein sequence databases used in traditional proteomic analyses. This cyclical process enhances the overall accuracy and efficiency of proteomic research (Created with BioRender.com, accessed on 1 April 2024).

Proteogenomics also strides towards personalizing medicine in nephrology. By integrating and analyzing patient-specific genomic, transcriptomic, and proteomic data, it allows for the customization of therapeutic strategies tailored to the unique molecular profile of an individual’s kidney disease [7,8,9,10]. This approach not only enhances the efficacy of treatments but also mitigates potential adverse effects, heralding a new era of patient-centric care in nephrology (Figure 1).

Our review introduces the concept of proteogenomics and its scientific significance in nephrology. It underscores the importance of this integrative approach in enhancing our understanding of kidney diseases and paves the way for subsequent chapters that dig deeper into specific applications and case studies of proteogenomics in kidney disease research.

This overview forms the foundation for further exploration into the multifaceted applications of proteogenomics in nephrology, setting the stage for a detailed examination of its role in advancing kidney disease research and patient care.

## 2. Methodological Framework of Proteogenomics

The proteogenomics pipeline embodies a methodical and integrated approach, intricately weaving together data from genomics, transcriptomics, and proteomics. This holistic methodology is pivotal in unraveling the complex interplay of genetic, transcriptomic, and proteomic variations, thereby offering a nuanced understanding of biological processes and disease mechanisms [11,12].

Genomic Data Integration: The pipeline initiates the collection and analysis of genomic data, which encompasses DNA sequencing to identify genetic variants, such as single nucleotide polymorphisms (SNPs), insertions, deletions, and structural variants. This genomic information provides the foundational layer for understanding genetic predispositions and alterations that may influence protein expression and function [13,14].

Transcriptomic Data Integration: Subsequent to genomic analysis, transcriptomic data is incorporated. This involves RNA sequencing (RNA-Seq) to quantify and characterize RNA transcripts. Transcriptomic analysis sheds light on the expression levels of genes and the presence of RNA splice variants, offering insights into how genetic variations translate into changes in mRNA expression [12,15].

Proteomic Data Integration: The proteomics component involves the use of mass spectrometry (MS) and other protein analysis techniques to profile the proteome. This stage aims to identify and quantify proteins, post-translational modifications, and protein–protein interactions. Proteomic data, when correlated with genomic and transcriptomic information, allows for the identification of novel proteins, splice variants, and protein isoforms that are relevant to disease states [7,12,16].

Data Analysis and Integration: The core of the proteogenomics pipeline lies in the sophisticated integration and analysis of data from these three domains. Advanced computational tools and bioinformatics approaches are employed to cross-reference and align data across these layers. This involves mapping proteomic data back to the genome and transcriptome, identifying correlations, and interpreting the functional implications of these associations [5,17].

Application in Disease Research: in the context of disease research, particularly in nephrology, this integrative approach facilitates a more precise identification of disease mechanisms, potential biomarkers, and therapeutic targets. It enables researchers to trace the path from genetic variation, through transcriptomic changes, to alterations in the proteome, thereby illuminating the molecular basis of kidney diseases [7].

In summary, the proteogenomics pipeline represents a confluence of multiomic data, harnessing the strengths of genomics, transcriptomics, and proteomics. Reflecting on the proteogenomic approach, we aim to elucidate how studies, even those focusing on single protein biomarkers, fit within the proteogenomic framework when integrated with genomic insights. This integrative and systematic approach is crucial in deepening our understanding of complex diseases, particularly in the field of nephrology (Table 1).

As we transition from discussing the theoretical underpinnings of the proteogenomics pipeline to exploring its practical applications in nephrology, it is pivotal to recognize that the integration of comprehensive proteomic analyses, such as LC/MS, with genomic data represents an emerging yet crucial frontier.

In the dynamic field of nephrology, proteogenomics stands as a promising avenue for uncovering the molecular intricacies of kidney diseases. However, it is important to note that the integration of comprehensive proteomic analyses, such as LC/MS, with genomic data—a hallmark of proteogenomics—remains an emerging frontier. Many of the studies we discuss employ targeted proteomic approaches, focusing on the identification of specific proteins of interest rather than exhaustive proteome mapping. This targeted approach often stems from the studies’ specific research questions or the current technological and financial constraints. Nevertheless, these studies represent crucial steps toward the ultimate goal of full proteogenomic integration, laying the groundwork for future research where both comprehensive proteomic techniques and genomic insights can be combined to unveil new layers of understanding in kidney disease pathophysiology and treatment. We acknowledge the evolving nature of proteogenomic methodologies and anticipate future advancements that will allow for more extensive integration of proteomic and genomic data in nephrology research.

## 3. Proteogenomic Breakthroughs in Nephrology: Case Study Insights

In the realm of nephrology, the application of proteogenomics has emerged as a transformative approach to understanding and treating kidney diseases [18,19]. This section explores a seminal study that epitomizes the use of proteogenomic analysis in the context of clear cell renal cell carcinoma (ccRCC). Several studies exemplified how integrating genomic, transcriptomic, and proteomic data can unravel the complex mechanisms underlying the response to cancer therapies, specifically tyrosine kinase inhibitors. Through this lens, we explore the intricacies of ccRCC, the nuances of treatment response, and the potential of proteogenomics in paving the way for personalized medicine in nephrology.

To clarify, the inclusion of renal cell carcinoma (RCC), especially clear cell renal cell carcinoma, in this review reflects its direct impact on renal function and pathology. Originating from renal tubular epithelial cells, ccRCC embodies the complex interplay between oncological development and kidney health, underscoring its relevance to nephrological research.

In exploring the impactful applications of proteogenomics in nephrology, this review highlights key studies, including those focused on ccRCC. While ccRCC is traditionally associated with urology–oncology, its relevance to nephrology cannot be understated. Renal carcinomas, by virtue of their origin, provide a unique window into the molecular dynamics of kidney health and disease. The intersection of oncological processes with renal function underscores the importance of a comprehensive approach to kidney disease research. Proteogenomics, by revealing the complex genetic and proteomic landscape of ccRCC, offers invaluable insights into the mechanisms driving kidney cancer and its systemic effects on renal physiology. These findings not only pave the way for novel therapeutic strategies but also enrich our understanding of kidney disease pathogenesis. The inclusion of ccRCC in our discussion serves to illustrate the broad applicability of proteogenomics in nephrology, encompassing both direct kidney ailments and related systemic conditions that affect kidney health.

For instance, the study “Proteogenomics of clear cell renal cell carcinoma response to tyrosine kinase inhibitor” offers a deep dive into the intricate dynamics of ccRCC and its response to the tyrosine kinase inhibitor, sunitinib [17]. This comprehensive analysis, integrating proteogenomic data, provides a clearer understanding of the molecular differences between patients who respond to sunitinib treatment and those who do not.

Key aspects of this study include the identification of molecular characteristics and pathways that correlate with treatment outcomes. Notably, the study reveals that chromosome alterations and specific signaling pathways, such as mTOR signaling, play a significant role in determining the response to sunitinib. Furthermore, the research highlights the diverse nature of the tumor microenvironment in ccRCC and its implications for treatment response.

One of the most groundbreaking outcomes of this research is the development of a multi-omics classifier. This tool can accurately differentiate between patients likely to respond to sunitinib and those who are not, paving the way for more personalized treatment strategies in ccRCC. The study also suggests that the integration of proteomic data with genomic and transcriptomic information can significantly enhance our understanding of cancer and treatment response, offering a potential model for future research in oncology.

The findings from this study have profound implications for the treatment of ccRCC and potentially other cancers. They underscore the importance of a personalized approach to cancer treatment, where understanding the individual molecular profile of a patient’s tumor can lead to more effective and tailored therapies. This approach could be a significant step forward in improving outcomes for patients with ccRCC and other complex cancers.

Another article, “Integrated Proteogenomic Characterization of Clear Cell Renal Cell Carcinoma” presents a comprehensive study that utilizes a proteogenomic approach to further understand ccRCC [20]. This in-depth analysis involves comparing genomic, transcriptomic, proteomic, and phosphoproteomic data from ccRCC tumors and normal adjacent tissues, providing a multi-layered view of the molecular alterations in ccRCC.

The study identifies key genomic alterations in ccRCC, such as chromosome arm-level changes and somatic mutations, and explores their impact on mRNA, protein, and phosphoprotein levels. This approach reveals novel insights into the dysregulated cellular mechanisms and pathways in ccRCC, driven by these genomic changes. For instance, the study highlights the altered expression of key proteins and pathways involved in metabolic processes, immune response, and cell signaling.

Additionally, the research delineates distinct immune-based subtypes of ccRCC, characterized by their specific cellular pathways and genomic alterations. These subtypes demonstrate the potential for more tailored therapeutic strategies, as they are associated with different clinical outcomes and responses to therapies.

This proteogenomic characterization of ccRCC underscores the complexity of cancer biology and the value of integrating multiple layers of molecular data. The findings from this study pave the way for future research in personalized medicine, offering new perspectives on treatment selection and patient care in ccRCC. The detailed exploration in this article showcases the significant strides being made in understanding and treating kidney cancer through the lens of proteogenomics.

Another example could be Autosomal Recessive Polycystic Kidney Disease (ARPKD), which is a type of kidney disease that is caused by mutations in the PKHD1 gene, located on chromosome 6p12. This gene plays a crucial role in the normal function of the renal tubular epithelium, a layer of cells that line the inside of the kidney’s tubules.

In our discussion on polycystic kidney disease, we aim to distinguish more clearly between Autosomal Recessive Polycystic Kidney Disease (ARPKD) and Autosomal Dominant Polycystic Kidney Disease (ADPKD). While ARPKD is indeed rare, making proteomic studies less common, ADPKD has seen emerging proteomic research. This distinction is crucial for understanding the specific pathological and clinical implications of each condition.

Despite the similarities in their names, ARPKD and ADPKD differ significantly in their genetic origins, progression, and clinical management. ARPKD, associated with mutations in the PKHD1 gene, typically presents earlier in life and involves distinct challenges in clinical management and treatment options. In contrast, ADPKD, often caused by mutations in the PKD1 or PKD2 genes, presents later and has been the focus of extensive proteomic research aimed at identifying therapeutic targets and biomarkers for disease progression. The emerging proteogenomic research in ADPKD offers hope for similarly advanced studies in ARPKD as technologies and methodologies evolve, potentially leading to more personalized and effective treatments for all forms of polycystic kidney disease.

The pathophysiology of ARPKD involves the abnormal proliferation of renal tubule epithelium, which can lead to the loss of their normal physiological function. This abnormal proliferation causes the kidneys to secrete fluid in the ducts, which are rich in epithelial growth factors. This leads to further proliferation of epithelial cells, resulting in the formation of cysts [21].

Proteogenomics, which combines proteomics and genomics, can provide valuable insights into the molecular mechanisms underlying ARPKD. For example, it can help identify changes in the expression of proteins encoded by the PKHD1 gene, which can provide clues about the development and progression of the disease. Furthermore, proteogenomics can also help identify potential therapeutic targets in ARPKD. For instance, animal models studying epithelial growth receptor blockers and epithelial enzymes have shown promising results, suggesting that targeting these proteins could be a viable therapeutic strategy for ARPKD [22].

When combined, proteomics and genomics can provide a detailed picture of the molecular mechanisms underlying ARPKD. For example, genomics can identify mutations in the PKHD1 gene, which is known to cause ARPKD. Proteomics can then be used to analyze the effects of these mutations on the proteins produced by the PKHD1 gene, providing insights into how these changes contribute to the pathophysiology of the disease [23].

Moreover, proteomics can identify changes in the expression of proteins in ARPKD patients, which can provide clues about the disease’s development and progression. For instance, the study of extracellular vesicles, which are released into the urine when cells are stressed, has shown that changes in the quantity or nature of released EVs may be related to the onset of the disease or the effectiveness of treatment [24,25].

To summarize, the integration of proteogenomic analysis in kidney disease studies offers a groundbreaking approach in nephrology. These studies highlight how combining genomic, transcriptomic, proteomic, and phosphoproteomic data can provide a comprehensive understanding of molecular alterations. This integrated approach not only reveals key insights into the mechanisms driving ccRCC and ARPKD but also paves the way for more personalized treatment strategies. The potential to tailor therapeutic approaches based on individual molecular profiles marks a significant advancement in the field, promising improved patient outcomes in kidney cancer treatment.

## 4. Advancements in Biomarker Discovery for Kidney Diseases

Advancements in utilizing proteogenomics biomarker discovery for kidney diseases have been significant, particularly in relation to CKD. These advancements have improved our understanding of the pathophysiological mechanisms of kidney damage and have the potential to enhance the clinical treatment of kidney diseases. They allow us to detect early damage, localize injury, and predict disease progression, severity, and associated long-term mortality [26].

One of the key advancements is the use of large, prospective CKD cohorts like the Salford Kidney Study, the German CKD Study, or the Chronic Renal Insufficiency Cohort Study. These studies provide an invaluable resource to identify patients whose pattern and rate of CKD progression can be accurately characterized using validated techniques. This allows for biomarker analysis in bio-banked samples during the course of patients’ CKD progression [27].

The rate of progression of CKD is often assessed by measuring the Glomerular Filtration Rate (eGFR), which indicates how well the kidneys are filtering blood. By defining the rate of eGFR change over time (ΔeGFR), we can better understand the progression of CKD. However, it is important to note that the progression can be non-linear and episodic, with phases of stability interrupted by periods of eGFR decline.

Proteogenomics has indeed identified several specific protein biomarkers in CKD. Among the most notable is *Complement C1q*: this protein was identified as a kidney-derived protein that could serve as a potential biomarker for CKD [28]. The researchers measured the amount of C1q in renal influx and efflux blood samples from seven individuals. They found that C1q was increased in the efflux samples from all individuals except one, indicating that C1q could be a candidate kidney-derived protein. Another one, - *uromodulin,* also known as Tamm–Horsfall protein, - is a glycoprotein expressed only by renal tubular cells. Studies have shown that its concentrations in CKD patients were lower than in healthy subjects, and the lower concentrations were associated with more advanced stages of CKD [29]. Uromodulin levels were positively associated with estimated GFR and inversely associated with proteinuria, as well as independently associated with End-Stage Renal Disease (ESRD) or rapid loss of estimated GFR [30].

Several other proteins besides Complement C1q and uromodulin have been detected as potential biomarkers for CKD through proteogenomics, each with unique implications for diagnosis, disease monitoring, and therapeutic strategies. Here are a few of them:

- *Cystatin C* is a C-type lectin, synthesized in all nucleated cells of the kidney, emerges as a superior marker for estimating glomerular filtration rate (GFR), with studies demonstrating its advantage over traditional creatinine-based methods in various patient populations (reference to meta-analysis or systematic review). Its levels, often unaffected by muscle mass, make it particularly valuable in assessing kidney function in elderly and pediatric patients. [31].
- *β-Trace Protein* (BTP) and *Kidney Injury Molecule 1* (KIM-1) are highlighted for their roles in evaluating renal tubular integrity. BTP’s utility extends beyond kidney function assessment to potentially pinpoint specific tubular injuries, while KIM-1 stands out in detecting acute kidney injury (AKI) transitioning to CKD, offering a prognostic marker for disease progression. Protein is produced by the proximal tubules of the kidney [32,33,34,35].
- *Neutrophil Gelatinase-Associated Lipocalin* (NGAL) is accentuated for its rapid response to kidney injury, serving as an early biomarker for AKI, with elevated urinary levels indicative of tubular damage before significant changes in GFR occur. This makes NGAL a critical tool for early intervention strategies [36,37].
- *Liver-Type Fatty Acid–Binding Protein* (L-FABP) and *Asymmetric Dimethylarginine* (ADMA) are discussed for their specific links to diabetic nephropathy and cardiovascular risks in CKD patients, respectively. L-FABP’s association with oxidative stress in diabetic nephropathy positions it as a marker for both diagnosis and monitoring disease severity. ADMA, by reflecting nitric oxide synthesis inhibition, offers insights into endothelial dysfunction, a common complication in CKD [38,39,40]
- Furthermore, the role of *microRNAs* in CKD is explored, emphasizing their potential as non-invasive biomarkers for disease detection and monitoring. Changes in microRNA profiles have been correlated with CKD progression and response to treatment, illustrating the dynamic nature of gene expression regulation in kidney disease pathogenesis [27,28,30].

In light of the evolving landscape of kidney disease research, it is pertinent to acknowledge the established role of biomarkers such as Cystatin C in the estimation of GFR, a cornerstone in the management of chronic kidney disease (CKD). While Cystatin C itself is not a novel biomarker, the advent of proteogenomics offers a unique opportunity to revisit and potentially recontextualize the utility of such established biomarkers. Proteogenomics, by enabling a more detailed examination of the genetic and proteomic underpinnings of biomarker expression and its correlation with disease states, holds the promise of refining our understanding of biomarker reliability and applicability across diverse patient populations.

These proteins represent just a small fraction of the potential biomarkers that could be identified through proteogenomics. As the field continues to advance, it is likely that even more proteins will be discovered that could serve as useful markers for CKD and other diseases.

Building upon the framework of proteogenomic biomarker discovery in CKD, our investigation further elucidates the relationship between key biomarkers and clinical parameters such as ACR and eGFR. The forthcoming table (Table 2) delineates this relationship, highlighting biomarkers identified through rigorous validation in both pilot and extensive patient cohort studies. Notably, significant correlations involving proteins like Adiponectin and Apolipoprotein A-IV underscore their diagnostic potential in CKD, thereby enriching our biomarker repository. These insights exemplify the depth of proteogenomic analysis in uncovering biomarkers that may revolutionize CKD diagnosis and management [41].

As the proteogenomic landscape continues to evolve, the translation of biomarker discoveries into clinical practice remains paramount. Each biomarker mentioned, from Adiponectin to Protein AMBP, represents not only a potential diagnostic tool but also a window into the underlying pathophysiological processes of CKD and its various manifestations, including diabetic kidney disease (DKD). The significance of these biomarkers demonstrated through their correlations with ACR and eGFR, as shown in Table 2, underscores their potential to inform clinical decisions, guide therapeutic interventions, and monitor disease progression. However, to fully leverage these biomarkers’ capabilities, it is crucial to understand their biological origins, the mechanisms by which they indicate kidney damage or disease progression, and their performance in clinical studies.

This understanding is fostered through comprehensive analysis and validation in diverse patient populations. Moving forward, our aim is to bridge the gap between biomarker discovery and clinical utility, ensuring that these proteins’ diagnostic and prognostic value is fully realized in patient care. The continued exploration and validation of these biomarkers will undoubtedly enrich our arsenal against CKD, enhancing our ability to predict, diagnose, and treat this complex disease more effectively.

## 5. Challenges and Solutions in Proteogenomic Analysis

The field of proteogenomic analysis, while offering groundbreaking insights into disease research, confronts several intricate challenges. One of the primary complexities lies in the integration of data across different omics platforms. The disparate nature of data from genomics, transcriptomics, and proteomics poses a challenge in achieving a cohesive analysis. Proteogenomics analysis involves studying the relationship between genes and proteins within an organism’s genome. It provides valuable insights into the role of proteins in various biological processes and diseases. However, there are several challenges associated with this field.

The complexity of the human proteome appears to be one of the main challenges. The human body contains tens of thousands of protein-coding genes and each one can produce multiple variants due to alternative splicing and post-translational modifications. This complexity makes it difficult to capture the full range of proteins expressed in a particular cell type or condition [42,43].

Another limitation is the difficulty in accurately quantifying individual proteins. Even with modern mass spectrometry techniques, there can still be considerable variability in the abundance of individual proteins. Additionally, the presence of degraded proteins and contaminants can further complicate the analysis.

Further complicating this landscape is the limitation of bioinformatics tools. The current tools vary in their capacity to handle the vast and diverse datasets generated in proteogenomic studies, often requiring significant customization and expertise. Proteogenomics involves analyzing large volumes of complex data. The data includes information about the genes present in the genome, the corresponding proteins, and the relationships between them. Handling and interpreting this data require advanced computational tools and techniques.

Quality and variability in data, especially in proteomics, also pose significant challenges. Proteomic data is inherently complex and can vary greatly due to experimental conditions, sample handling, and the sensitivity of detection methods. This variability necessitates stringent quality control measures and robust analytical approaches to ensure the reliability of the findings.

Developing efficient algorithms for proteogenomics analysis is another challenge. These algorithms need to accurately identify proteins from mass spectrometry data, match these proteins to their corresponding genes in the genome, and predict their roles in biological processes. Implementing these algorithms efficiently and accurately is a significant task.

However, there is a lack of standardized software for proteogenomics analysis. Different research groups may use different software tools, which can lead to inconsistencies and difficulties in comparing results across studies. The sheer volume of data generated requires extensive computational resources, along with specialized knowledge in data processing and analysis. This can be addressed through advancements in computational tools and techniques, algorithm development, and software standardization:

1. Use of Advanced Computational Tools: Leveraging high-performance computing and cloud computing platforms can help manage the large volume of data generated in proteogenomics analysis. For example, Hadoop MapReduce and Amazon Web Services have been used to run X!Tandem in parallel on collections of commodity computers [44].
2. Development of Efficient Algorithms: Using advanced algorithms like MCtandem for large-scale peptide identification on many integrated core (MIC) architecture can enhance the efficiency of proteogenomics analysis [45,46,47]
3. Standardization of Software: Establishing standards for proteogenomics software can ensure consistency across different research groups. This could involve creating common interfaces or formats for input and output data [13].

While high-end software solutions could be costly, there are several open-source tools available for proteogenomics analysis. Here are a few notable ones:

1. Peptide Spectrum Matching (PSM) Tools: These tools are used to match experimental spectra with theoretical spectra to identify peptides. Examples include PGTools, Galaxy-P, ProteoAnnotator, IPAW, JUMPg, Graph2Pro/Var2Pep, NextSearch, and PGP. These tools support execution on distributed memory environments using job scheduling frameworks like PBS or Torque [1,46,48].
2. Peptide Mapping Tools: These tools map discovered peptides to the genome. Examples include GenoSuite, Enosi, Bacterial Proteogenomic Pipeline, and MSProGene. Some of these tools only use a single execution core, while others support multi-core processing [49,50].
3. PGA: This is a proteogenomics analysis tool available on GitHub. It allows you to perform de novo peptide sequencing and protein inference from tandem mass spectrometry data [47,51].

These tools can handle different aspects of proteogenomics analysis, including genomics processing, proteomics filtering, peptide-spectrum matching, false discovery rate (FDR) analysis, and peptide-to-genome mapping. They offer a range of functionalities and can be used in different combinations depending on the specific requirements of the analysis.

Furthermore, the interpretation of proteogenomic data requires sophisticated bioinformatics tools and algorithms. While there have been advances in this area, there are still many challenges remaining, including the need for more accurate and robust algorithms, the development of standards for data sharing and validation, and the integration of proteogenomic data with other omics data [43].

Finally, the cost and time required for proteogenomic experiments can be prohibitive. High-throughput sequencing technologies are expensive and require significant computational resources for data analysis. Furthermore, the process of generating proteogenomic data involves multiple steps, including sample preparation, protein extraction, labeling, and data acquisition, which can be time-consuming.

Addressing these challenges requires a multifaceted approach. Development and standardization of advanced bioinformatics tools are critical in simplifying data integration and interpretation. Enhancing the quality of proteomic data through improved methodologies and standardization protocols is essential for consistent and reliable results. Collaborative efforts across disciplines, combining expertise in biology, bioinformatics, and computational science, are vital in overcoming these barriers, thereby propelling the field of proteogenomics toward more significant discoveries and applications in disease research.

Despite these challenges, proteogenomics holds great promise for the study of kidney diseases and other complex conditions. Continuous advancements in technology and bioinformatics, along with increased collaboration among researchers, will likely help overcome these limitations in the future.

## 6. Clinical Implications and Future Directions

Proteogenomics, the study of proteins and genomes, holds significant potential in the field of CKD. It has been used in several studies related to kidney diseases, although the specific examples provided in this review are limited.

1. Diabetic Nephropathy: In a study published in the Journal of American Society of Nephrology, urinary proteomics was used to analyze the impact of diabetes on kidney function. The researchers found that diabetes significantly affected the urinary proteome, suggesting that it could serve as a potential biomarker for diabetic nephropathy [52].
2. Chronic Kidney Disease (CKD): Another study, also published in the Journal of American Society of Nephrology, explored the use of urinary proteomics in the diagnosis and monitoring of CKD. The researchers used a technique called Collision Energy Mass Spectrometry (CE-MS) to analyze the human urinary proteome, aiming to discover biomarkers for CKD and other kidney diseases [53].
3. Aristolochic Acid Toxicity: Aristolochic acid is a poisonous substance that can cause kidney failure. A study published in the journal, Kidney International, used proteogenomics to investigate the toxic effects of aristolochic acid on cultured renal epithelial cells. The researchers found that the drug caused significant changes in the proteome of the cells, indicating its potential as a therapeutic target [54].

These examples illustrate how proteogenomics can be used to study various kidney diseases, from diabetic nephropathy to chronic kidney disease. However, it is important to note that proteogenomics is a complex field with many challenges and more research is needed to fully realize its potential in the study of kidney diseases.

Generally, proteogenomics could provide valuable insights into the genetic and protein alterations that occur in the kidneys due to CKD, potentially leading to improved diagnostic and therapeutic strategies. This approach combines genomics (studying genes) and proteomics (studying proteins) to provide a more comprehensive understanding of disease mechanisms.

However, without specific studies or findings related to CKD, it is difficult to provide a more detailed explanation. For instance, understanding the specific proteins and genes involved in CKD could lead to the development of targeted therapies or personalized treatments. Additionally, integrating proteomic data with other omics data (like genomics, transcriptomics, and metabolomics) could further enhance the precision of CKD diagnosis and management.

Future directions might involve developing new technologies for proteogenomic analysis, creating databases for CKD-related proteins and genes, and conducting large-scale proteogenomic studies in CKD patients. These efforts could pave the way for more accurate and personalized treatment plans for CKD patients.

## 7. Conclusions

In this review, we aimed to provide a comprehensive overview of the field of proteogenomics and its transformative potential in nephrology, focusing on broad concepts rather than specific case studies. This approach was deliberately chosen to emphasize the wide-ranging applications and theoretical implications that proteogenomics holds for advancing our understanding of kidney diseases, catering to a diverse readership.

Instead of detailing specific examples of proteogenomic applications, we encourage readers to explore the references cited throughout this review. These references are carefully selected to guide those interested in delving into detailed examples of how proteogenomics is being successfully applied in the field of nephrology. While this review primarily focuses on the application of proteogenomics in rare and complex kidney diseases, the principles and techniques discussed may also hold significant potential for enhancing diagnostic and therapeutic strategies in more common kidney diseases. Future research could beneficially explore these applications. By seamlessly integrating genomic, transcriptomic, and proteomic data, proteogenomics has illuminated the complexities of kidney diseases at a molecular level. This has led to the discovery of novel biomarkers and the development of personalized treatment strategies, significantly enhancing patient outcomes. However, challenges such as data integration complexities and the need for advanced bioinformatics tools remain. Looking ahead, the continuous evolution of proteogenomic methodologies promises to further revolutionize nephrology, driving us towards a future where personalized medicine is the norm and where the treatment of kidney diseases is more precise and effective. The journey of proteogenomics in nephrology is just beginning, and its full potential is yet to be realized.

## Figures and Tables

**Table 1:** The depicted framework provides a comprehensive view of the typical data layers encountered in proteogenomic research. It initiates with patient-specific clinical profiles, encapsulating a spectrum of characteristics that can range from basic identifiers to extensive phenotypic details. Subsequently, the framework progresses to delineate the genetic blueprint of individuals, capturing a vast array of both inherited and mutation-derived genetic markers through high-throughput sequencing techniques. Following the genetic overview, the narrative extends to encompass gene activity patterns, including the transcriptome’s coding and regulatory sequences, potentially cataloging tens of thousands of unique molecular signatures. The culmination of this hierarchical structure is the exploration of the proteome, delving into the abundance and diverse post-translational modifications of proteins, where the data spans from a few thousand to a substantial catalog of tens of thousands of protein features.

	Observable Traits	Methods/Technique	Category of Data	Rough Count of Attributes
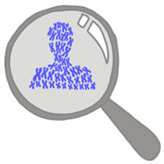	Phenotype	Clinical Metadata	Sample Annotation	10–100
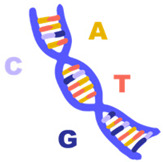	DNA	Whole Exome Sequencing Whole Genome Sequencing	Germline mutations Somatic Mutations CNA	10–20 K 20–30 K 15–20 K
		Methylation Array	DNA methylation	15–20 K
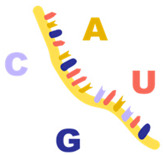	RNA	RNA-seq	mRNA Circular RNA	15–20 K
		miRNA-seq	miRNA	2–3 K
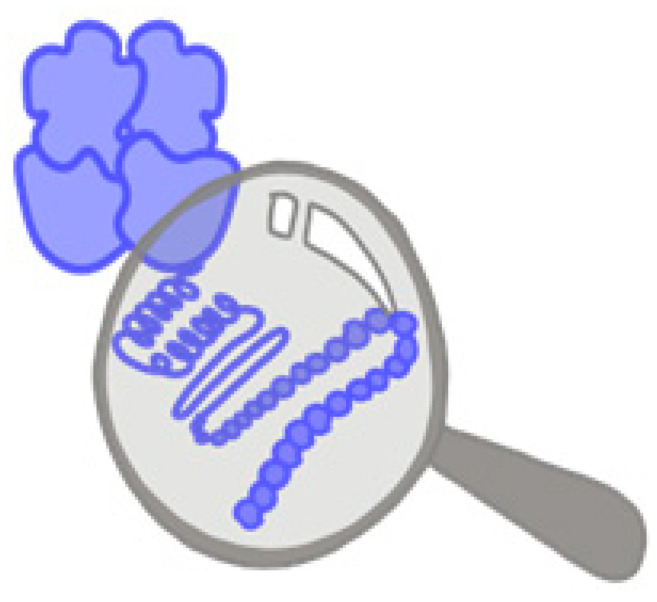	Protein	LC proteomics	Proteins Phosphorylation Acetylation	10–15 K 30–50 K 5–10 K
		MS proteomics	Ubiquitylation Glycosylation	10–20 K 10–15 K

**Table 2:** This table summarizes the correlation of selected biomarkers with ACR and eGFR in the context of diabetic kidney disease (DKD) [41]. Data are derived from both a pilot study involving 30 patients and a comprehensive validation study with 572 patients. *p*-values indicate the statistical significance of correlations, with bold values highlighting significant correlations (*p* < 0.05). This analysis underscores the potential of these biomarkers in diagnosing and monitoring DKD progression.

Protein Name	Peptide	Pilot Study ACR *p*-Value	Validation Study ACR *p*-Value	Validation Study eGFR *p*-Value
Adiponectin (ADIPO)	GDIGETGVPGAEGPR	0.008	0.251	0.089
Apolipoprotein A-IV (APOA4)	LEPYADQLR, ISASAEELR	>0.1 0.083	0.002 <0.001	<0.001 <0.001
Apolipoprotein C-III (APOC3)	DALSSVQESQVAQQAR	0.056	0.701	0.004
Complement C1q subcomponent subunit B (C1QB)	IAFSATR	0.002	0.063	0.382
Complement factor H-related protein 2 (CFHR2)	TGDIVEFVCK LVYPSCEEK	>0.1 0.030	0.090 0.010	<0.001 <0.001
Hemoglobin subunit beta (HBB)	SAVTALWGK	0.052	<0.001	0.355
Insulin-like growth factor-binding protein 3 (IBP3)	VNVDEVGGEALGR, ALAQCAPPPAVCAELVR	0.052 0.083	<0.001 <0.001	0.346 0.060
Protein AMBP (AMBP)	FLNVLSPR, TVAACNLPIVR	0.069 >0.1	<0.001 0.017	0.019 0.049
